# The Research Family Advisory Committee: the patient and family view of implementing a research-focused patient engagement strategy

**DOI:** 10.1186/s40900-022-00335-z

**Published:** 2022-02-05

**Authors:** Francine Buchanan, Amy Peasgood, Megan Easton, Karen Haas, Unni Narayanan

**Affiliations:** 1 Research Family Advisory Committee, Hospital for Sick Children Research Institute, The Hospital for Sick Children (SickKids), 555 University Ave, Toronto, ON M5G1X8 Canada; 2 Child Health Evaluative Sciences, The SickKids Research Institute, Toronto, Canada; 3 Division of Orthopaedics, The Hospital for Sick Children (SickKids), Toronto, Canada; 4 Division of Orthopaedic Surgery, Department of Surgery and Rehabilitation Sciences Institute, University of Toronto, Toronto, Canada

**Keywords:** Patient engagement, Patient perspective, Patienta: Patient engagement in research, Paediatrics

## Abstract

Patient engagement in research, a collaborative practice of including patients and families as active and respected partners in the research process, leads to improved quality of patient care and positively affects outcomes for patients and families. There is strong support for the benefits of patient engagement. What is less clear are the methods by which organizations can achieve authentic patient engagement in research and the ways a committee structure can support an institutional research engagement need beyond the individual investigators. In this report, we describe the mechanisms needed to support the implementation of a research-focused patient engagement strategy and lessons learned from the patient and family perspective.

## Plain English summary

Patient engagement in research is a way of working in partnership with patients and members of the public to plan, manage, design and carry out health research, leading to improved patient care. Extensive research has been published on the practices individual research teams can use to positively engage patients and families in research. A current gap in existing research is the methods health care organization can use to achieve authentic patient engagement. In this report, we describe the implementation of a research-focused patient engagement strategy and the lessons learned from the patients and families involved in developing the strategy.

## Introduction and background

In 1948, Kurt Lewin, a social psychologist, said that research “needs the best of what the best among us can give and the help of everybody” [1]. Dr. Lewin’s words would foresee the increase in wider stakeholder participation in health research, now coined as patient engagement in research. Patient engagement in research, a collaborative practice of including patients and families as active and respected partners in the research process [2, 3], provides for greater opportunities to impact the ‘triple aim’ of patient-centred care [4], which is to improve the patient experience of care (including quality and satisfaction); improve the health of populations; and reduce the per capita cost of health care [5].

To improve the quality of patient care and positively affect outcomes for patients, recent research into patient engagement demonstrates the need for active patient involvement in the development and support of research, service delivery and outcome evaluation [2, 4, 6]. There is strong support for the benefits of patient engagement and the values of institutions, researchers and organizations that support it [2, 4]. What is less clear are the methods by which organizations can achieve these goals to maximize patient engagement and the ways a committee structure can support an institutional need beyond the individual investigator [3]. In this report, we describe the mechanisms needed to support the implementation of a research-focused patient engagement strategy and lessons learned from the patient and family perspective. In 2016, we initiated the Research Family Advisory Committee (RFAC) for patient and family engagement in research at the Hospital for Sick Children.

## Supportive structures

**Fig. 1:**
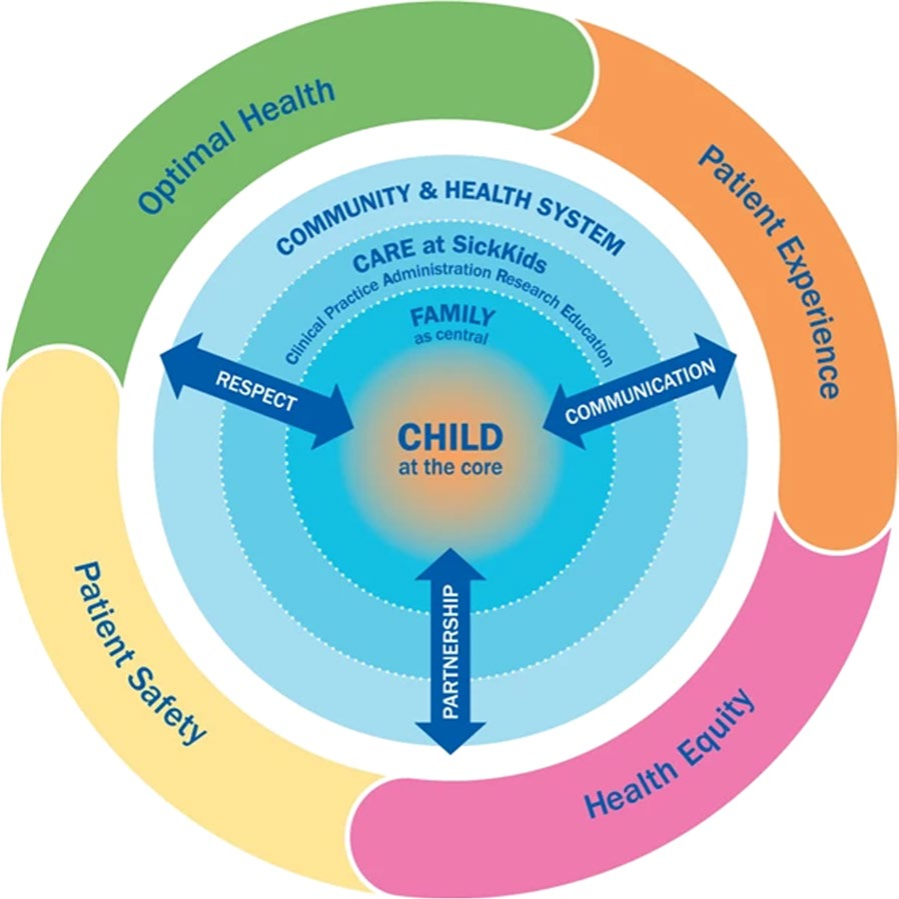
SickKids' model of child and family-centred care

Prior to implementing a research patient engagement strategy, the Child and Family Centred Care model was already implemented and active in engaging family input into the development of hospital services [7, 8]. This model of care places the patient and family at the centre of healthcare and recognizes the child and family as key systems to be engaged with, rather than entities that health care practitioners ‘do for’ in the delivery of paediatric care [7]. Core components of the model include dignity and respect; information sharing and communication; and participation [7, 8] (Fig. 1).

The aim of developing the child and family centred care model was to promote a shared understanding of child and family centred care for those within the institution and to inform the design, implementation, and evaluation of clinical practices, research, administrative support, and educational initiatives within the SickKids community [8].

To facilitate the mandate of child and family-centred care and raise the voice of the patient and family, a number of committees were formed, including the Family Advisory Network (FAN), Children’s Council and The Family-Centred Care Advisory Council (FCCAC). These committees meet regularly and are supported through the Centre for Innovation & Excellence in Child and Family-Centred Care.

The FCCAC and the Children’s Council were formed to facilitate family engagement and representation in clinical encounters. Members of the FCCAC and the Children’s Council are recruited from a larger group of patients and families that comprise the Family Advisory Network (FAN). The FAN is comprised of approximately 100 patients and family members, who advise on short-term projects in the hospital (e.g., patient safety, quality improvement, patient care, facilities planning) or serve on committees in specific clinical programs, such as Haematology/Oncology, Neonatal Intensive Care and Critical Care. Prospective patients and family members interested in joining the family advisory network complete a formal application, undergo screening and participate in a SickKids volunteer onboarding process, which is an important step as it provides context to the families as they participate in hospital functions and are educated on policies—especially privacy, infection control, and safety training. Although the FAN, FCCAC and Children’s Council were resources available to researchers, those groups focused on clinical implementation work and did not have an understanding of the research process or how to meet the unique needs of researchers and the staff of the Research Institute (RI).

Investigators began adopting patient and family-friendly research practices along with increased adoption of the patient-oriented research practices promoted by the Canadian Institutes of Health Research (CIHR) and the Strategy for Patient-Oriented Research (SPOR). Individual research teams instituted their own family advisory teams [9] and developed tools that included patients’ perspectives to develop and use patient-reported outcome measures [10]. The SickKids RI, however, did not have an organizational process to recruit and access patient and family advisors to enable the hundreds of researchers that lacked the resources, time, or knowledge to develop their own group of family advisors. There was also a strong desire from research faculty and staff to understand how to best incorporate input from patient and family advisors and to set consistent expectations on how to engage with patients and families on research studies.

## Finding a research-specific solution

To increase the adoption of child- and family-centred practices [7] within the research community, and increase the inclusion of patient perspectives in research [2, 4, 6], a multidisciplinary group of research coordinators, clinician-scientists, administrators, patients and family representatives of the SickKids Family Advisory Network (FAN) was assembled. The diverse composition was an important aspect in developing the objectives. The goal of the Patient Engagement in Research Secretariat Working Group (PEiR-WG) was to advance patient and family-oriented research at SickKids by developing policies, tools and practices that promoted the active engagement of patients and families throughout the research process. The overarching goal was to identify and implement evidence-based solutions that would meet the needs of researchers, patients and families to increase inclusion of the child and family perspective in the design, implementation, and dissemination of clinical research.

A systematic method of identifying membership included ensuring that members of the group were comprised of individuals who had the following characteristics:

- Former patients who had experience being a research study participant, and
- Family members that were familiar with the research process and had experience as advisors on research studies.
- Researchers who had practical experience conducting patient-oriented research, or
- Researchers who had experience conducting research but self-identified as lacking in knowledge on how to engage patients in research.

The mindful selection of group members supported the group’s aims of developing solutions that were practical to implement, based on the real needs of users, and guided by existing research on patient and family engagement. To ensure the PEiR-WG was grounded in values underlying patient engagement practices, journal articles, guidance documents, and values statements were circulated from authoritative sources in patient and family engagement and patient-oriented research [2, 11]. A library of resources was compiled with the support of a SickKids staff librarian and made accessible to all members.

The SickKids PEiR-WG met on a bi-monthly basis, with work completed between meetings. In meetings, team members generated ideas for projects to be advanced, assessing them for the relationship to the RI’s strategic plan, ease of implementation, the requirement for resources, and potential for impact. To support the working group’s decision-making, a review of research engagement practices was conducted and gaps identified. A gap identified was support for pediatric researchers embarking on engaging patients for the first time. The PEiR-WG identified a priority project to create a research-focused family advisory group that would serve as a resource for RI staff looking for input from patient and family advisors. The PEiR-WG made recommendations on the composition of the advisory group and developed a framework for how the group should be supported, ensuring it was accountable to the PEiR-WG members. Foundational to the development of what would become the SickKids Research Family Advisory Committee (RFAC), was that the time invested by both committee members and research teams seeking guidance from the team was used wisely. It was important to the members of the PEiR-WG that the RFAC advisors be able to see how their input was incorporated into the conduct of studies, feel comfortable providing input in meetings, and be provided with enough information to provide meaningful input.

## Launching and sustaining the Research Family Advisory Committee (RFAC)

The RFAC was officially launched in 2018 based on the functions and responsibilities outlined by the PEiR-WG (Table 1). The mandate of the RFAC upon launch was to advance patient- and family-oriented research at SickKids by facilitating the active engagement of patients and families throughout the research process. The intent of the RFAC is to provide SickKids researchers an efficient means of accessing an already assembled group familiar with a wide range of patient engagement practices to support the development or execution of a study. Members of the RFAC were recruited from internal postings to RI staff and existing FAN membership. All members were asked to submit nominations outlining their background, experience, and interest in joining the RFAC. All nominations were vetted by a select group of PEiR-WG members, senior leadership sponsors, and the patient and family coordinator from the Centre for Innovation and Excellence in Child & Family-Centred Care. Members were selected to represent a broad range of roles within the RI, backgrounds within different fields of study, clinical settings, and healthcare experiences. In total the inaugural group of RFAC members consisted of eight FAN members, eight RI staff, two co-chairs from the FAN and RI staff respectively, and two executive sponsors. The group was also supported by administrative support staff that consisted of the patient and family engagement coordinator, the project manager leading the PEiR-WG, and a research program coordinator. The RFAC met in person on SickKids premises, once a month for 2 h with meetings starting at 6:00 pm to accommodate members who could not attend daytime meetings due to other commitments. For those unable to attend in person, a teleconferencing option was made available. Each meeting followed a pre-specified agenda that included one or two presentations by researchers seeking RFAC advice, typically 45 min each to allow for rich discussions; with the remaining time allocated to updates on prior RFAC activities; discussion of new RFAC priorities and projects; or presentations of educational content for the RFAC. Since April 2020, these meetings have continued virtually using video-conferencing with the same level of engagement. The RFAC and PEiR-WG continue to run simultaneously with the PEiR-WG continuing to focus on developing policies, tools and practices while the RFAC focus remain on engaging with research teams to provide the patient perspective to specific research studies.

**Table 1:** RFAC goals and objectives

1. Promote the active engagement of patients and families, including but not limited to research advisors, throughout the research process including:
(a) Identification of research priorities
(b) Development of research ideas
(c) Development of research methodology
(d) Review of research findings
(e) Dissemination of research findings
2. Provide input on research proposals, including their child and family-centeredness and their strategies for patient and family engagement
3. Facilitate the placement of research advisors on research teams
4. Offer educational opportunities to research advisors to enhance their understanding of the research process
5.. Offer educational offerings to researchers on the role of research advisors on research teams
6. Provide forums for dialogue between patients, families, and researchers
7. Promote awareness of the value of research and participation in research among patients and their families
8. Establish standards related to high-quality patient and family engagement in research

## Lessons learned and continuous improvement

From the onset, a guiding principle of the RFAC has been to retain membership by ensuring each attendee is valued and each meeting provides a return on each RFAC member’s investment of time. Outcomes from continuous dialogue on ways to improve the experiences of RFAC members are documented, refined and shared with all members (Tables 2, 3). Based on the low turnover of members (only three members have resigned since RFAC initiation), maintaining a focus on value has been successful.

“As a parent member, I feel like I’ve evolved in step with the whole committee. Since we were all founding members, it’s been a mutual learning experience. Every time we meet, we discover something new that works or doesn’t, and we adjust accordingly. There’s a great spirit of continuous improvement overall” (RFAC parent member).

**Table 2:** RFAC conditions for success

Factors that influence engagement	Conditions for success
*The patient* Individual factors that can affect patients’ motivation, willingness, and ability to engage within and across different levels 1. Patients’ knowledge, attitudes, and beliefs 2. Experience with the health care system 3. Self-efficacy 4. Functional capacity	Ensure a diverse mixture of membership considering background, experiences, education, culture, language, and expertise Schedule meetings in the evenings in an accessible location to accommodate members who have commitments during the day Offer a variety of means to attend the meeting (e.g. in-person, online virtual, or telephone) Provide various means of contributing to the discussion (e.g. online chats, email, post-meeting collaborative forums, verbal debriefs) Ensure volunteer members are compensated for any costs for participation (e.g. parking, public transit costs) Acknowledge and value diverse backgrounds, perspectives, and ideas by openly discussing and debating input. Do not dismiss ideas without providing your reason and rationale
*The Organization* An organization’s characteristics influence patients’ ability to engage in it Staff practices Organizational policies or practices	Devote appropriate resources to support the group to minimize the amount of time members must spend on RFAC duties outside of meeting times. Examples of support tasks include: Identifying and preparing presenters Providing facilitation notes to chairs Prioritizing pre-reading material Collecting feedback from presenters Promote an environment of continuous improvement, including regularly reflecting on what is working well and what needs re-thinking. Address areas for improvement with actionable ideas Ensure staff and volunteers are equally informed and updated on organizational changes or updates Promote a culture of continuous learning by providing opportunities for training and development of knowledge and skills related to research, communication. and co-learning
*Society* Patients and organizations operate within a broader social and political environment and are influenced by social norms; regulations; and national, provincial, and local policies	Promote a culture of openly addressing larger cultural issues (e.g. Isolation from COVID-19 restrictions) and how they may affect participation in RFAC duties Ensure appropriate resources are engaged to adapt quickly to larger societal or organizational impacts

**Table 3:** Presentation tips to RFAC presenters

Only include in the presentation the background information required to make the discussion valuable
Spark the discussion using targeted questions that address components of your study you can change. (e.g. ​We are considering using focus groups as a data collection method. Have you participated in a study using a focus group? What do you believe are the pros and cons of being part of a focus group?)
Consider using breakout rooms to facilitate more targeted discussions
Consider using an online collaboration tool such as ideaboardz or a MS teams (This allows for RFAC members to contribute ideas or notes after the meeting as ended)

This list was developed by RFAC members as part of a continuous improvement process

The internal resources to support the RFAC have been integral to sustaining the committee and ensuring the goals of the committee are being met. Outside of the monthly meetings, support staff invest an additional 20–40 h per month collectively in meeting preparation, identifying researchers, seeking input, and ensuring presenters are informed of what to expect at the RFAC meeting. The support staff and co-chairs have a minimum of two meetings before each RFAC to set the meeting agenda. This includes identifying, prioritizing, and vetting potential research presenters; preparing training materials to present; and reviewing feedback from presenters as to how RFAC input has been incorporated into studies.

“From the time of onboarding, there have been consistent and meaningful opportunities for enriching my knowledge of patient partnership in research. Whether the leaders are sharing online learning modules, original RFAC-developed content, or directing us to formal courses on the subject, they make it easy to deepen our understanding based on whatever time we have available” (RFAC parent member).

A key practice that has added value to the RFAC meeting as well as improved the experience for both RFAC members and presenters is a one-hour meeting held between the co-chairs and each presenter two weeks prior to the RFAC meeting. The meeting is designed to prepare presenters for presentations. In this meeting, presenters are asked to explain their study, outline the questions they plan to pose to RFAC members and discuss their expectations on feedback. Through these discussions, the RFAC co-chairs guide the presenters to focus the questions posed to the RFAC on areas in which changes can be made. Since the launch of the RFAC, pre-meetings have evolved and based on learnings, a pre-meeting guide has been developed to guide the conversations (Table 3). The main area that has been beneficial in preparing presenters is to ensure that questions posed to the RFAC are targeted, focused on specific issues that are actionable by the research team. To ensure this, teams are asked to inform the RFAC of any areas of the study which cannot be altered, such as language within questionnaires based on validated measures, or methods that may be dictated by industry partners or partner sites. The investment in pre-meetings has ensured that presentations are focused only on sharing the material necessary to inform input. Whenever possible, based on the outcome of the preparation meeting, pre-reads are sent out to RFAC members at least one week prior to the monthly meeting to ensure that RFAC members have enough information to adequately contribute informed feedback.

“As a parent, I’ve found membership on the RFAC to be rewarding in multiple ways. I’ve connected with other parents who share my commitment to partnership in research, gained a deeper awareness of researchers’ challenges and priorities, and been inspired to become a more informed partner by taking a course on family engagement in research” (Family Advisor, RFAC member).

## Discussion and conclusion

Ensuring the success and sustainability of a research-specific patient and family advisory committee requires appropriate planning, resources, a flexible mindset, readiness for change, and a firm grounding in agreed-upon values. Considering and agreeing to these elements before the development of a program, and continually improving and refining the process, will ensure that the program meets the needs of its target audience and team members. From the onset, the culture of the RFAC was to be inclusive with diverse ideas and various backgrounds and experiences. In order to promote continuous engagement of patient and family perspectives, and ground the RFAC in the values of patient-centred care, patient and family voices were recognized as important and valuable. Elevating the visibility of patient and family voices was accomplished by storytelling around advisory experiences and providing opportunities for researchers to spend time with advisors. Crucially important for the RFAC is that researchers’ efforts to facilitate patient and family engagement in their research are genuine and meaningful, rather than tokenistic. The RFAC plays a significant role in the education of researchers in this regard.

"I think RFAC is incredibly valuable because I will be one of the research "tools" in my project as the interviewer of families. Therefore, it is incredibly important that I understand how parents perceive my questions...Having the feedback of the RFAC allows me as an interviewer to be more aware, sharpening the "tool" in this project" (Clinician-Scientist, RFAC Presenter).

The impact of the RFAC include increased awareness amongst researchers on the role patient and family voice have on shaping research practices, integration of family friendly language into patient facing study materials, and anecdotal reports of increase study participation. Research teams with no prior experience working with patient and family advisors have reported that their experience with the RFAC has motivated them to include patient and family engagement practices in future study design.

A key driver of the success of the RFAC has been the Institution’s commitment to provide the operational administrative support for the RFAC and for its members. The mutual benefit gained from all members was invaluable, and the opportunity to learn from colleagues across a wide breadth of research also supported the advisors’ desire to grow. All of these factors have built a strong foundation for a sustainable resource critical to improving the quality of clinical research conducted at the hospital.

## Data Availability

Not applicable.

## Abbreviations

CIHR: Canadian Institutes of Health Research
FAN: Family Advisory Network
FCCAC: Family Centred Care Advisory Council
PEiR-WG: Patient Engagement in Research Secretariat Working Group
RFAC: Research Family Advisory Committee
SPOR: Strategy for Patient-Oriented Research
